# Physician–nurse conflict resolution styles in primary health care

**DOI:** 10.1002/nop2.1147

**Published:** 2021-12-14

**Authors:** Barbara Delak, Klemen Širok

**Affiliations:** ^1^ Community Health Centre Ljubljana Ljubljana Slovenia; ^2^ University of Primorska ‐ College of Health Care Izola Slovenia

**Keywords:** conflict, conflict resolution styles, nurses, physicians, primary health care

## Abstract

**Aim:**

To examine the conflict resolution styles used in the physician–nurse teamwork in primary health care, depending on individual characteristics, contextual factors, and organizational and sociocultural contexts.

**Background:**

Conflicts significantly affect the quality of healthcare services and staffing challenges, and consequently the performance and efficiency of organizations. Their management plays an important role in any healthcare organizations and deserves the attention of researcher's healthcare management and team leaders.

**Design:**

A descriptive, cross‐sectional, correlational design.

**Method:**

Thomas–Kilmann Conflict MODE Instrument was used on a sample comprising 173 nurses and 125 physicians working in teams at the Community Health Centre Ljubljana, Slovenia, in 2018.

**Results:**

The most predominant conflict resolution styles were compromising and avoiding, followed by accommodating, collaborating and competing. The predominant resolution style among nurses was avoiding, and among physicians was compromising. There were statistically significant differences in the conflict resolution style according to gender (*χ* ^2^ (1) = 0.035, *p* < .059), education (*χ* ^2^ (1) = 0.014, *p* < .05) and tenure (*χ* ^2^ (1) = 0.025, *p* < .05).

**Conclusion:**

Our research results differed from those of other studies, possibly due to the difference in the sample and research setting. They indicated that the specifics of work and situation significantly moderate conflict in healthcare organizations.

**Impact:**

The established divergence of results indicates the need for future research on conflict in healthcare settings to more consistently consider the situational context and the role of management and/or leadership.

## 1 INTRODUCTION

In complex healthcare organizations, conflicts significantly affect the quality of healthcare services and staffing challenges; therefore, they deserve attention from researchers and practitioners. Nurses and physicians are key occupational groups for healthcare provision, and their effective collaboration, to a large extent, affects healthcare performance and efficiency. A review on conflict in the healthcare domain presents extensive and rather dated research on conflict management styles between staff nurses and nurse managers; however, no research has been conducted yet on conflict resolution styles in the physician–nurse relationship or in primary health care.

## 2 BACKGROUND

The literature review showed declining research interest in the role of conflict in the healthcare setting over past 10 years. Al‐Hamdan et al. (2011) stated that nurses comprise the largest healthcare professional group and are routinely confronted with complex problems involving conflicts among staff and patients. Furthermore, nurses and physicians differ in their professional roles and therefore often face collaboration challenges (Kantek & Yesilbas, 2020). Conflict may emerge as a result of several elements such as the complexity of an organization, varying role expectations, interdepartmental competition, constraints in the decision‐making process, competition over limited resources, unclear job boundaries and personality differences (Patton, 2014). Thus, conflict management becomes an integral and essential aspect of healthcare organizational activity (Al‐Hamdan et al., 2011). Appropriate conflict management and conflict resolution improve cooperation between nurses and physicians, which, in turn, increase the satisfaction of all stakeholders involved in the healthcare process, including patients. It also improves the quality of health care (Al‐Hamdan et al., 2011) and patient outcomes (Hendel et al., 2007), while reducing costs, which consequently increases the organization's performance and efficiency (Skjørshammer, 2001). Moreover, an untreated conflict is expensive for healthcare organizations because it wastes time and money, and leads to employee turnover (Slaikeu & Hasson, 2012). Unresolved conflicts may have several negative effects on patient outcomes, loyalty to the organization and work commitment (Almost et al., 2010). Additionally, different approaches to conflict management may affect staff job satisfaction (Kunaviktikul et al., 2000), job performance (Shih & Susanto, 2010) and intention to stay (Almost et al., 2010).

There is an empirical gap about research on conflict resolution among healthcare professionals in primary health care, and between nurses and physicians, who often work collaboratively in teams. In their review, Labrague et al. (2018) found that nine studies compared conflict management styles between staff nurses and nurse managers, while the remaining three articles compared nurses' styles of managing conflict with those of other healthcare professionals. They also noted that only three studies were from the European Union. Related to this was Al‐Hamdan et al. (2011) finding of different cultures having different conflict resolution styles. This research gap deserves attention; after all, primary health care is the cornerstone of health systems and a cost‐effective way towards universal health coverage (World Health Organization, 2020).

## 3 THE STUDY

### 3.1 Aim

The main aim of this exploratory study was to examine the conflict resolution styles used by nurses and physicians in primary health care when collaborating in teams, depending on individual characteristics, contextual factors, and organizational and sociocultural contexts. The physician–nurse perspective in primary healthcare obtained from this study has been used to highlight the need for further research in this field and to inform the key personnel of healthcare organizations—including team leaders—on importance of conflict management.

### 3.2 Study design and participants

A descriptive, cross‐sectional, correlational design was used. The research was carried out between March–April 2018 in the largest primary healthcare centre in Slovenia (Community Health Centre Ljubljana) among 850 units: 476 nurses (56%) and 374 physicians (44%) working in teams, which were all sent the invitation to survey. 422 units responded (49% response rate), and 298 fully and correctly returned surveys were obtained: 173 from nurses (58.1%) and 125 from physicians (41.9%). The survey instructions clearly indicated that the respondents should consider the situations pertaining to physician–nurse teamwork.

A comparison of the population and respondents' structure showed that the sample adequately reflected the structure of the population (Table 1). The professional role, gender and managerial position structures were comparable. There were some differences in educational and tenure structures, with those of longer tenure (20–25 years) and undergraduate 1st cycle being over‐represented, and MMSc being under‐represented.

**TABLE 1:** Comparison of personnel structure with respondents' structure

Demographics	Population *N* = 850	% of the population	Respondents N = 298	% of the respondents
Professional role				
Nurse	476	56.0	173	58.1
Physician	374	44.0	125	41.9
Gender				
Male	63	7.4	24	8.1
Female	787	92.6	274	91.9
Educational level				
Vocational secondary education (3 years)	11	1.3	6	2.0
Professional secondary education (4 years)	299	35.2	85	28.5
Higher vocational education	26	3.1	22	7.5
Undergraduate 1st cycle	136	16.0	95	31.9
Master in nursing or master of medicine (MMSc)	356	41.8	80	26.8
MSc	13	1.5	7	2.3
PhD	9	1.1	3	1.0
Tenure				
Up to 5 years	124	14.6	32	10.7
5–10 years	144	16.9	28	9.4
10–15 years	118	13.9	25	8.4
15–20 years	69	8.1	21	7.0
20–25 years	87	10.2	65	21.8
25–30 years	55	6.5	0	0.0
30–35 years	120	14.2	55	18.5
Over 35 years	133	15.6	72	24.2
Managerial position				
Yes	73	8.6	36	12.1
No	777	91.4	262	87.9

### 3.3 Data collection

This study was supported by the Healthcare Center Management. Web surveying was used for data collection, more specifically the LimeSurvey, wherein all population units received an email with the invitation letter stating the purpose of the study, the participants' right to confidentiality, their voluntary participation and the link to the survey. Participants were also given the contact information of the primary researcher in case they had any questions about the study. The anonymity of the respondents and confidentiality of the information provided were ensured throughout the study. During the data collection period, two reminders were sent to the non‐respondents.

### 3.4 Ethical considerations

Since the research does not have a direct impact on people, no approval from an ethics committee was sought.

### 3.5 Data analysis

Using SPSS version 24, the collected data were inspected, processed into a ranking scale and then analysed followed the established methods of analysing the results of the MODE Instrument (Thomas, 1974). As mentioned earlier, the five conflict management styles evaluated by this instrument are not independent, and add up to a constant; hence, a comparison of averages between individual groups was not possible. For data analysis, we used univariate analysis through frequency distributions and descriptive statistics, and bivariate analysis to test hypotheses based on the chi‐squared (*χ* ^2^) test. The level of statistical significance was set at *p* < .05.

### 3.6 Validity, reliability and rigour

The validated Slovene translation of “Thomas–Kilmann Conflict MODE Instrument” (Šubic, 2017; Thomas, 1974) was used. It includes five individual styles of resolving a conflict: collaborating, accommodating, avoiding, competing and compromising. It should be noted that in practice, all individuals, whether leaders or not, habitually use only a limited number of styles (perhaps just one) to resolve all the conflicts in which they are involved (Kilmann & Thomas, 1977). The questionnaire contains 30 pairs of statements about possible responses in a particular conflict situation. Respondents must choose the statement that describes them the best. This form of forced choice between the two statements, in addition to limiting social bias, also eliminates the usual biased responses involved in Likert scales, such as leniency and rigour (Kilmann & Thomas, 1977). A higher score in one style means a corresponding reduction in scale points in the other styles, which is why the five styles are not independent. The conflict resolution style that has the highest score is the most pronounced style in the individual.

The MODE instrument was chosen because of the comparability of its results and its high levels of validity and reliability. Kilmann and Thomas (1977) reported the following test–retest correlation coefficients for the instrument's individual styles after 4 weeks: competing, 0.61; collaborating, 0.63; compromising, 0.66; avoiding, 0.68; and accommodating, 0.62. Volkema and Bergmann (1995) determined a test–retest correlation coefficient of 0.77. Kilmann and Thomas (1977) calculated an average Cronbach's alpha coefficient of 0.60, establishing its internal reliability.

## 4 RESULTS

Predominant conflict resolution styles of participants' groups are listed below in Table 2. In our sample of physician–nurse teams, compromising (44.3%) and avoiding (42.3%) dominated as conflict resolution styles, followed far behind by accommodating (7.7%), collaborating (3.4%) and competing (2.3%). The predominant conflict resolution style of nurses was avoiding and that of physicians was compromising, whereby no statistically significant differences (*χ* ^2^ (1) = 0.844, *p* < .05) were found based on respondents' professional role. The predominant style of conflict resolution among nurses was avoiding (44.5%), followed by compromising (42.8%), accommodating (7.5%), collaborating (3.5%) and competing (1.7%). The opposite was true for physicians: their predominant style was compromising (46.4%), followed by avoiding (39.2%), accommodating (8.0%), collaborating (3.2%) and competing (3.2%).

**TABLE 2:** Results of predominant conflict resolution styles

Characteristics	Competing	Collaborating	Compromising	Avoiding	Accommodating
Observed participants, *N* (%)	7 (2.3)	10 (3.4)	132 (44.3)	126 (42.3)	23 (7.7)
Professional role, *N* (%)					
Nurses	3 (1.79)	6 (3.5)	74 (42.8)	77 (44.5)	13 (7.5)
Physicians	4 (3.2)	4 (3.2)	58 (46.4)	49 (39.2)	10 (8.0)
Gender, *N* (%)					
Man	2 (8.3)	2 (8.3)	14 (58.3)^*^	5 (20.8)	1 (4.2)
Women	5 (1.8)	8 (2.9)	118 (43.1)	121 (44.2)^*^	22 (8.0)
Level of education, *N* (%)					
Vocational secondary education (3 years)	0 (0.0)	0 (0.0)	4 (66.7)^*^	0 (0.0)	2 (33.3)
Professional secondary education (4 years)	0 (0.0)	3 (3.5)	34 (40.0)	41 (48.2)	7 (8.2)
Higher vocational education	1 (4.5)	2 (9.1)	10 (45.5)	9 (40.9)	0 (0.0)
Undergraduate 1st cycle	3 (3.2)	2 (2.1)	36 (37.9)	46 (48.4)	8 (8.4)
Master in nursing or master of medicine (MMSc)	2 (2.5)	1 (1.3)	45 (56.3)	26 (32.5)	6 (7.5)
MSc	1 (14.3)	1 (14.3)	3 (42.9)	2 (28.6)	0 (0.0)
PhD	0 (0.0)	1 (33.3)	0 (0.0)	2 (66.7)^*^	0 (0.0)
Tenure, *N* (%)					
Up to 5 years			23 (76.7)^*^	7 (23.3)	
5–10 years			17 (68.0)^*^	8 (32.0)	
10–15 years			8 (38.1)	13 (61.9)^*^	
15–20 years			8 (50.0)	8 (50.0)	
20–25 years			28 (49.1)	29 (50.9)^*^	
25–30 years			0 (0.0)	0 (0.0)	
30–35 years			18 (43.9)	23 (56.1)^*^	
Over 35 years			30 (44.1)	38 (55.9)^*^	
Managerial position, *N* (%)					
Yes	1 (2.8)	1 (2.8)	19 (52.8)	14 (38.9)	1 (2.8)
No	6 (2.3)	9 (3.4)	113 (43.1)	112 (42.7)	22 (8.4)

^*^Statistically significant, *p* < .05.

We found statistically significant differences by gender (*χ* ^2^ (1) = 0.035, *p* < .05), wherein men mostly chose compromising (58.3%) over avoiding (20.8%), and women preferred avoiding (44.2%) slightly more than compromising (43.1%).

We also observed statistically significant differences in conflict resolution styles according to the attained level of education (*χ* ^2^ (1) = 0.014, *p* < .05), wherein those with a vocational secondary education (3 years) preferred compromising (66.7%), while those with a PhD mostly chose the avoiding style (66.7%). The compromising style was prevalent among respondents with higher vocational education (45.5%), master in nursing or master of medicine (MMSc) (56.3%), and a MSc (42.9%), while the avoiding style prevailed in respondents having completed professional secondary school (4 years) (48.2%) and those with a undergraduate 1st cycle (48.4%).

Furthermore, longer tenure was significantly related to the predominant conflict resolution style. The initial chi‐squared test showed that there were no statistically significant differences based on tenure in the predominant conflict resolution style (*χ* ^2^ (1) = 0.059, *p* > .05). However, since the remaining styles were weakly represented, we observed only the two predominant styles of conflict resolution (compromising and avoiding) and found statistically significant differences according to tenure (*χ* ^2^ (1) = 0.025, *p* < .05). Those with a tenure of up to 10 years chose the compromising style in most cases (up to 5 years, 76.7%; 5–10 years, 68.0%), while those with a tenure of more than 10 years (except those with 15–20 years, 50.0%) chose avoiding (10–15 years, 61.9%; 20–25 years, 50.9%; 30–35 years, 56.1%; and over 35 years, 55.9%).

When observing the managerial position of the respondents, there were no statistically significant differences (*χ* ^2^ (1) = 0.709, *p* < .05) in the preferred conflict resolution style. Nurses and physicians in a managerial position preferred the compromising (52.8%) style more than those in non‐managerial positions (43.1%). The second most preferred style in both groups was avoiding (managers, 38.9%; non‐managers, 42.7%). Other styles of conflict resolution were rarely chosen.

## 5 DISCUSSION

The results represent an interesting starting point for reflection, since our research showed large discrepancies when compared to other conflict studies in the field of nursing. It should be noted that a comprehensive review of the literature uncovered only three studies that were comparable to our research in terms of the research goals (Akel & Elazeem, 2015; Hendel et al., 2007; Kaitelidou et al., 2012), wherein studies specifically addressing conflict resolution in primary health care were not found. Next, each of the major findings is first compared with the results of studies carried out in similar research settings (primary healthcare) and/or with similar samples (conflict resolution styles in physician–nurse teamwork). Then, we make a brief comparison of the results with those of other studies that explore conflict resolution styles in health care and finally provide the hypothetical explanation of the results.

Our findings were similar to those of two studies conducted in similar research settings: avoiding was the most frequently used resolution style by nurses and physicians in paediatric hospitals in Greece (Kaitelidou et al., 2012), and compromising was the most common resolution style among both professional groups in hospitals in Israel (Hendel et al., 2007). Accommodating was the most predominant style used by nurses and physicians in Egypt (Akel & Elazeem, 2015); additionally, the compromising style was used significantly more often by physicians than by nurses in this study sample. In the five studies with different research settings, similar findings were identified, namely the use of avoiding by nurses (Johansen & Cadmus, 2016; Morrison, 2008) and staff nurses (El Dahshan & Keshk, 2014), and compromising by head nurses (Hendel et al., 2005) and nurses (Iglesias & de Vallejo, 2012). However, six other studies reported avoiding as the least frequently used conflict management style among nurse managers in Oman (Al‐Hamdan, 2009; Al‐Hamdan et al., 2011), Turkey (Kantek & Kavla, 2007) and Egypt (Mohamed & Yousef, 2014), and among nurses in Israel (Tabak & Orit, 2007) and Finland (Ylitörmänen et al., 2015).

Considering gender, our results also represent a significant deviation from other studies. Akel and Elazeem (2015) observed the dominating style to be a frequently used strategy for men in both professional groups. Three studies with a different research setting, one in Iran and two in Oman, also found male nurses to compromise more often than their female nurse co‐workers (Al‐Hamdan, 2009; Al‐Hamdan et al., 2011). In contrast, female nurse managers in Oman and Jordan tended to use the avoiding style (Al‐Hamdan, 2009) and integrating style, respectively. Male nurse managers were also found to use the avoiding style in a study by Al‐Hamdan et al. (2014). Three other studies identified the divergent styles of integrating, obliging and competing to be the most frequently used styles among male nurses in handling conflict (Al‐Hamdan et al., 2016; El Dahshan & Keshk, 2014; Kaitelidou et al., 2012).

Considering education, our results differ from those of the three studies conducted in similar research settings, but correspond to those found in Finland and Turkey. Avoiding conflict situations was a more common style among paediatric nurses with a undergraduate 1st cycle than among nurses with a higher vocational education (Ylitörmänen et al., 2015). Supervisors and nurses in Turkey with an higher vocational education adopted the avoiding and competing styles more than the other styles (Tuncay et al., 2018). We also found diverging results in two studies with different research settings (Al‐Hamdan et al., 2011; Başoğul & Özgür, 2016).

Our study also provided a completely new picture of differences in conflict resolution style based on tenure. The compromising style was more often used by those with tenure of up to 10 years, while everyone else with a longer tenure preferred to use the avoiding style.

Considering managerial position, we did not find any statistically significant differences; that is the participants preferred to compromise rather than avoid irrespective of whether they were employed in a managerial position, meaning that there were no comparable findings among studies with similar research settings. Studies conducted in different research settings also showed divergent results.

An explanation for the passive (submissive) behaviour of nurses can be found in occupational stratification and occupational status. We notice that there is still a very strong occupational differentiation and professionalization among physicians (profession) and nurses (semi‐profession) in Slovenia. When comparing the nursing occupation with physicians and other healthcare professions in terms of the salary, working conditions, and public and political influence, nursing is considered a semi‐profession (Witz, 1990). In past 30 years, physicians in Slovenia had 8 strikes that resulted in 2‐wage increases, whereby other healthcare workers only had 2 unsuccessful strikes. Another important aspect in this regard is that of values that are deeply rooted in a traditional system such as healthcare and can propagate submissive behaviour. In Slovenia, it is still common for physicians to treat nurses as second‐class members of the “same” team (Klemenc & Pahor, 2004). The value system of health professionals is largely formed during schooling, when candidates internalize the beliefs of their teachers, who prepare them for the profession (Rešetič, 2010). Last but not the least, "avoiding conflict" is also rooted in Slovene culture. As Gunkel et al. (2016) observe, cultural value dimensions affect conflict‐handling style preferences through the moderation of emotional intelligence.

Our observations about the predominant use of the avoiding style by nurses and the compromising style by physicians also coincide with the findings of other researchers. Skjørshammer (2001), and Tabak and Orit (2007) explain that the traditional paradigm of the physicians' role as leaders and the role of other healthcare professionals as dependent on them also reflects in conflict resolution. Furthermore, interpersonal relationships among healthcare professionals are often overlooked, pushed to the sidelines and perceived as less important and problematic due to the focus on treatment and nursing (i.e. patients). Consequently, nurses and physicians are more willing to compromise or avoid (Skjørshammer & Hofoss, 1999). Nurses and physicians are overworked, and do not have or take out the time to talk about interpersonal relationships and conflicts (Skjørshammer, 2001).

Some findings of our study can also be considered worrying. Those with a shorter tenure clearly want to resolve conflicts with moderate assertive behaviour and cooperation. In contrast, those with a longer tenure prefer to avoid conflicts, showing little concern for their own needs and those of others by leaving conflicts unresolved. Since longer tenures build work experience, more cooperation would be expected from such professionals. However, those with a longer tenure clearly realize that conflicts are better avoided. One of the reasons for this could be the hierarchical structure of organizations. Those with a shorter tenure, although initially more assertive, develop an apathetic attitude towards conflict resolution and display less (or no) commitment to assert their views because of time spent in the rigid healthcare system, which has strong values and an established organizational culture that does not encourage autonomy in thinking.

The question remains whether the observed empirical differences can be explained by the differences between primary and secondary health care. As this has not yet been empirically verified in Slovenia and, to our knowledge, elsewhere either, we can offer only a few hypothetical explanations, which are based mainly on our own observations arising from many years of work in the healthcare system. The cooperation between nurses and physicians in healthcare organizations at the primary healthcare level is much more intensive and continuous than at the secondary level, that is in hospitals, at least in Slovenia. As Brown et al. observe (2011), the demands and expectations on primary healthcare teams differ from specialty teams, such as stroke rehabilitation or oncology where care plans can be very disease‐specific and the role of team members more clearly delineated, amplifying the potential for conflict in teams.

### 5.1 Limitations

Our study employed convenience rather than random sampling, which might have resulted in sampling error and affected the generalizability of the results. In addition, the present study used a cross‐sectional design instead of a longitudinal design, as it was conducted only in one country and one institution with rather nationally homogenic teams. Thus, the research findings can be generalized only to select public healthcare institutions at the primary healthcare level in Slovenia. There might also be some other important factors that our research design could not control for. Another limitation might be the relatively small sample of male respondents and rather large share of uncompleted questionnaires that did not appear to generate systematic bias. Despite these limitations, the present results can still be considered reliable, and the study provides new insights about conflict management styles in primary health care.

## 6 CONCLUSIONS

Before presenting the conclusions, let us emphasize again that the goal of our research was different from the majority of studies that address and explain the emergence of conflict resolution styles among nurses. We specifically addressed the issue of predominant conflict resolution styles in teamwork settings between nurses and physicians in primary health care. Although the existing studies have shown divergent results related to different organizational and contextual factors, our study stands out in many respects. Cultural specifics play an important role in conflict resolution (Gunkel et al., 2016), and conflict resolution styles are also situationally dependent. For instance, Al‐Hamdan (2009) found that conflict management styles significantly differed according to hospital type and years of experience. If we evaluate the obtained results through our own perceptions and observations, formed on the basis of many years of work in health care, we can conclude that the empirical findings adequately reflect the actual situation and that contextual factors such as occupational status, values, organizational structure and situational context play an important role in conflict resolution in health care. Hence, the results of this study indicate that the specifics of work and situation significantly moderate conflict in healthcare organizations.

The established divergence in the results of comparable studies and, above all, the obvious discrepancies shown in our research results clearly indicate the need for future research on conflict in health care to more consistently consider the situational and organizational context in which conflicts arise. After all, only three of the studies we referred to involved physicians. However, none of them examined conflict resolution in physician–nurse collaborations, which obviously play an important role in ensuring quality health care and effective teamwork, and perhaps call into question the existing research findings.

Last but not least, despite its key importance in ensuring social welfare, primary health care does not receive adequate research attention, at least in the area of conflict research. First, patient health care begins at the primary level, where the majority of healthcare services are provided. Second, at least in Slovenia, the number of healthcare professionals at the primary level represents a third of all healthcare workers, which is certainly not negligible. Third, primary health care involves constant and intensive collaboration between nurses and physicians, whereas at the secondary level, this collaboration is not so continuous. Constructive conflict resolution is especially important during the time of the ongoing coronavirus pandemic, which has created extremely unpredictable and unfavourable situations. In particular, healthcare organizations need to be as flexible as possible and ready for rapid change, and effective collaboration between healthcare teams resulting from timely conflict resolution will be crucial for managing this situation successfully and effectively.

### 6.1 Implications

Variability of empirical results and the obvious role of contextual factors clearly signal the need for future research on conflict resolution in health care that should pay more attention to social and organizational context factors as moderating variables. Dealing with conflicts at the institutional level should be holistic, aimed at multiple sources of conflict through multiple approaches and supported by key stakeholders—especially team leaders and management.

Leadership style and choice of conflict management strategies may strongly influence conflict outcomes (Brown et al., 2011; Hendel et al., 2005). For effective management of conflict, leaders must pose right qualities and skills and understand the causes, approaches and strategies of conflict management. In addition, they need to adapt the use of these styles to the ongoing social circumstances (Thomas & Kilmann, 1975) and avoid becoming chronically committed to any one strategy. Studies identified specific characteristics of leaders as facilitating conflict resolution such as being accessible, non‐judgemental and employing good‐listening skills (Brown et al., 2011), open and direct communication, willing to find solutions, showing respect and the practice of humility (Çınar & Kaban, 2012). Also, the application of the appropriate conflict management style also depends on the change management lead by chief physicians and hospital managers, increasing the moral and motivation of the personnel (Çınar & Kaban, 2012). After all, the single most adaptable and powerful influence on the culture of modern organizations is leadership (West et al., 2014).

While our study observed conflict resolution strategies, sources of conflict should also be considered, when thinking what should be focus of the conflict management (West et al., 2014). While sources of conflict in primary health care have been researched in various papers, understanding of the potential barriers at micro (individual), meso (organizational) and macro (system) level is also important.

Strategies to address various sources of conflict are well known. Brown et al. (2011) recommend the use of conflict resolution protocols. Others (Çınar & Kaban, 2012; Hendel et al., 2005) recommend various kinds of trainings aiming at various skills development covering relevant organizational behaviour topics: conflict resolution strategies, decision‐making, negotiations, implementation of power and effective communication. Learning in the work environment can also be done through observations (Hendel et al., 2005). As observed in the previous section, preparation in conflict management should be included along the professional socialization process. Finally, true leaders should serve as role models in effective conflict resolution.

## CONFLICT OF INTEREST

The authors declare they have no financial interests.

## ETHICAL APPROVAL

Research Ethics Committee approval was not required as we had confirmation of management of Community Health Centre Ljubljana to conduct survey and to use data for publishing an article.

## Data Availability

The data that support the findings of this study are available on request from the corresponding author. The data are not publicly available due to privacy or ethical restrictions.
